# Warped graphitic layers generated by oxidation of fullerene extraction residue and its oxygen reduction catalytic activity

**DOI:** 10.3762/bjnano.10.137

**Published:** 2019-07-12

**Authors:** Machiko Takigami, Rieko Kobayashi, Takafumi Ishii, Yasuo Imashiro, Jun-ichi Ozaki

**Affiliations:** 1 Graduate School of Science and Technology, Gunma University, 1-5-1 Tenjin-cho, Kiryu, Gunma 376-8515, Japan; 2 R & D Center, Nissinbo Holdings Inc, 1-2-3 Onodai, Midori-ku, Chiba 267-0056, Japan

**Keywords:** carbon alloy catalysts, fullerene extraction residue, oxygen reduction reaction (ORR), polymer electrolyte fuel cells, warped graphitic layers

## Abstract

Carbon-based oxygen reduction reaction (ORR) catalysts are regarded as a promising candidate to replace the currently used Pt catalyst in polymer electrolyte fuel cells (PEFCs); however, the active sites remain under discussion. We predicted that warped graphitic layers (WGLs) are responsible for the ORR catalytic activity in some carbon catalysts (i.e., carbon alloy catalysts (CACs)). To prove our assumption, we needed to use WGLs consisting of carbon materials, but without any extrinsic catalytic elements, such as nitrogen, iron, or cobalt, which effectively enhance ORR activity. The present study employed a fullerene extraction residue as a starting material to construct WGLs. The oxidation of the material at 600 °C exposed the WGLs by removing the surrounding amorphous moieties. Transmission electron microscopy (TEM) observations revealed the formation of WGLs by oxidation treatment at 600 °C in an O_2_/N_2_ stream. Extending the oxidation time increased the purity of the WGL phase, but also simultaneously increased the concentration of oxygen-containing surface functional groups as monitored by temperature programmed desorption (TPD). The specific ORR activity increased with oxidation up to 1 h and then decreased with the intensive oxidation treatment. Correlations between the specific ORR activity and other parameters confirmed that the development of the WGL and the increase in the O/C ratio are the competing factors determining specific ORR activity. These results explain the maximum specific ORR activity after 1 h of oxidation time. WGLs were found to lower the heat of adsorption for O_2_ and to increase the occurrence of heterogeneous electron transfer.

## Introduction

Polymer electrolyte fuel cells (PEFCs) are used as the power supply for automobiles and stationary devices. Cost reduction, specifically the cost reduction of cathode catalysts, is imperative to apply PEFCs for practical use [1]. Increasing the specific activity of platinum catalysts is the most realistic approach; for example, by creating alloys [2] or core–shell structures [3] or by activating the Pt particles through metal support interactions [4]. Developing non-precious-metal catalysts is a fascinating, but ultimately speculative, technology for material scientists. Many studies have reported on the preparation of active non-precious catalysts; however, few mention the principles of the catalytic activity. All the studies reporting on the ORR activity suggest dependence on the following three principles: (1) formation of M–N_4_ surface complexes (M = Fe, Co) and its analog on carbon substrates [5–8]; (2) change in the electronic distribution by doping with nitrogen and other elements [9–22]; and (3) activation of the carbon surface by encapsulated metal particles [23–28].

Our non-precious-metal ORR catalysts are based on carbon alloy catalysts (CACs) [29]. CACs are carbon-based catalysts with active sites consisting of mainly carbon atoms. The sites are constructed by controlling the crystallographic and chemical states of carbon atoms through careful carbonization. Controlling carbonization by metal catalysts such as iron or cobalt produces nanoshell-containing carbon (NSCC) with ORR activity [30–35]. This activity is thought to originate from surface defects formed on the nanoshell carbons, including edges and warped graphitic layers (WGLs) [31,36]. Improving an ORR catalyst by altering the catalyst design and preparation successfully led to the world's first commercialization of a portable fuel cell with a non-precious-metal catalyst [37–38]. Building on this success, we ultimately aim to apply our CACs to automobile and stationary device uses. These applications require more active and more durable catalysts. The identification of the active sites of these CACs is an important issue for improving their activity and durability.

Active CACs commonly include WGLs [31]. We consider WGLs to be the basic structure responsible for the ORR activity of CACs. The ORR activity of the WGLs was examined using onion-like carbon (OLC) produced by the heat treatment of a nanodiamond [39]. The results showed the highest ORR activity for OLC heat-treated at 1400 °C among the prepared samples (HTT = 1000 °C to 1800 °C). The material formed OLCs composed of WGLs, but also included untransformed diamond. The residual diamond prevented us from confirming that WGLs are the active sites of the CACs.

The ideal carbon material to confirm our assumption that WGLs are responsible for the ORR active sites should not include foreign atoms, known as promoters, such as a diamond phase, nitrogen, boron, phosphorus, sulfur, and transition metals like iron and cobalt. Such WGLs can be obtained from fullerene-related materials. We selected a commercial carbon, Nanom Black, which is a fullerene extraction residue from a fullerene soot prepared by a combustion method [40]. The combustion method produces a large amount of fullerenes by partial thermal oxidation of hydrocarbons. The residue is essentially amorphous but should include WGLs containing a non-benzenoid structure due to some incomplete formation of fullerenes [41–42].

Our previous study on extracting nanoshell structures from a nanoshell-containing carbon (NSCC) by using H_2_O_2_ oxidation showed that extracting oxidation works well to produce WGLs from Nanom Black [43]. Here, we describe the formation of WGLs from Nanom Black by oxidation and show that the ORR activity of the obtained WGLs is related to their development in the material. Finally, we show that the WGLs are responsible for the catalytic activity of CACs.

## Experimental

### Sample preparation

Nanom Black (NB-ORG) is a residual carbon found after extracting fullerenes (e.g., C_60_ and C_70_) from a fullerene soot produced by a combusting method. It is commercially available from Frontier Carbon Inc. (Japan). From inductively coupled plasma atomic emission spectroscopy (ICP-AES) measured by Shimadzu Techno-Research, Inc., it can be concluded that the carbon possesses no ORR promoting metal elements. NB-ORG was heat-treated at 600 °C in an oxygen-containing stream (O_2_ [Vol.]/N_2_ [Vol.] = 6:94) for a duration ranging from 0.5 to 5 h. The oxidized NBs are referred to as ONB-*t* (*t* = 0.5, 1, 2, 3, 5) according to their oxidation time. A control was also prepared from NB-ORG by heating it at 600 °C in a nitrogen stream for 2 h (NNB).

### Characterization techniques

The structure of the prepared carbons was studied by transmission electron microscopy (TEM) and X-ray diffraction (XRD). The transmission electron microscope (JEM-2010, JEOL Inc.) was operated at an accelerating voltage of 200 kV. The X-ray diffractometer (XRD6100, Shimadzu Corp.) was equipped with a Cu Kα X-ray source (40 kV, 30 mA) and was operated by scanning the diffraction angles from 5° to 90° at a scanning speed of 1 °/min. Corrections for atomic scattering, Lorentz, and polarization factors [44] were made to the diffraction profile for detailed analysis of the 002 diffractions.

An automatic surface area analyzer (BELSORP MINI, Microtrak BEL Inc., Japan) was used to measure the N_2_ adsorption isotherms at 77 K after evacuating the sample at 200 °C for 2 h under a dynamic vacuum. The Brunauer–Emmett–Teller (BET) theory was applied to determine the surface area (BET-SSAs) of the samples as calculated from the isotherms. An automatic static adsorption analyzer (BELSORP Max, Microtrac BEL Inc., Japan) was used to measure the O_2_ adsorption isotherms at −80 °C with a pressure range of 5 to 100 kPa. A differential scanning calorimeter (DSC8500, Perkin-Elmer) was used to measure the heat of O_2_ adsorption of the carbons, and was operated by monitoring the heat-flux change when the stream was switched from nitrogen to oxygen. A laboratory-constructed temperature programmed desorption (TPD) apparatus recorded the TPD spectra of H_2_O, CO, CO_2_, and H_2_. The spectra were used to calculate the amount of surface functional groups. The details of this technique are described elsewhere [45–46].

### Electrochemical techniques

Cyclic voltammetry was used to evaluate the heterogeneous electron transfer rate of the carbons in an aqueous solution consisting of 6 × 10^−3^ mol/L K_3_[Fe(CN)_6_] and 1 mol/L KNO_3_. The carbons, bound by using a polyvinylidene fluoride (PVDF) resin solution (KF polymer L, #7305, NMP solution, Kureha Co. Ltd.) with a composition of carbon/PVDF = 1/0.5, served as a working electrode with round shape. A potentiostat system (ALS700 series, BAS Japan) was used to record the cyclic voltammograms in a potential range of 0.6 to −0.1 V vs Ag/AgCl at different sweep rates (1 to 50 mV/s).

Linear sweep voltammetry with a rotating disk electrode was used to evaluate the ORR activity of the prepared carbons. The carbons were used to form a working electrode by using a Nafion (Nafion perfluorinated resin solution, Aldrich) solution on a 4 mm glass-like carbon disk electrode (0.2 mg [carbon]/cm^2^, 0.1 mg [binder]/cm^2^). The reference electrode was a reversible hydrogen electrode (RHE). The electrolyte was a 0.5 mol/L H_2_SO_4_ aqueous solution. A voltammogram obtained in a N_2_-saturated electrolyte was subtracted from a voltammogram in an O_2_-saturated electrolyte giving a net ORR voltammogram. The electrochemical system consisted of a rotation apparatus (RRDE-3A, BAS Japan) and a potentiostat (ALS700 series, BAS Japan). The potential sweep was started from 1 V to 0 V vs RHE, at a potential sweep rate of 1 mV/s and a rotating speed of 1500 rpm.

## Results

### Carbon structure

**Figure 1:**
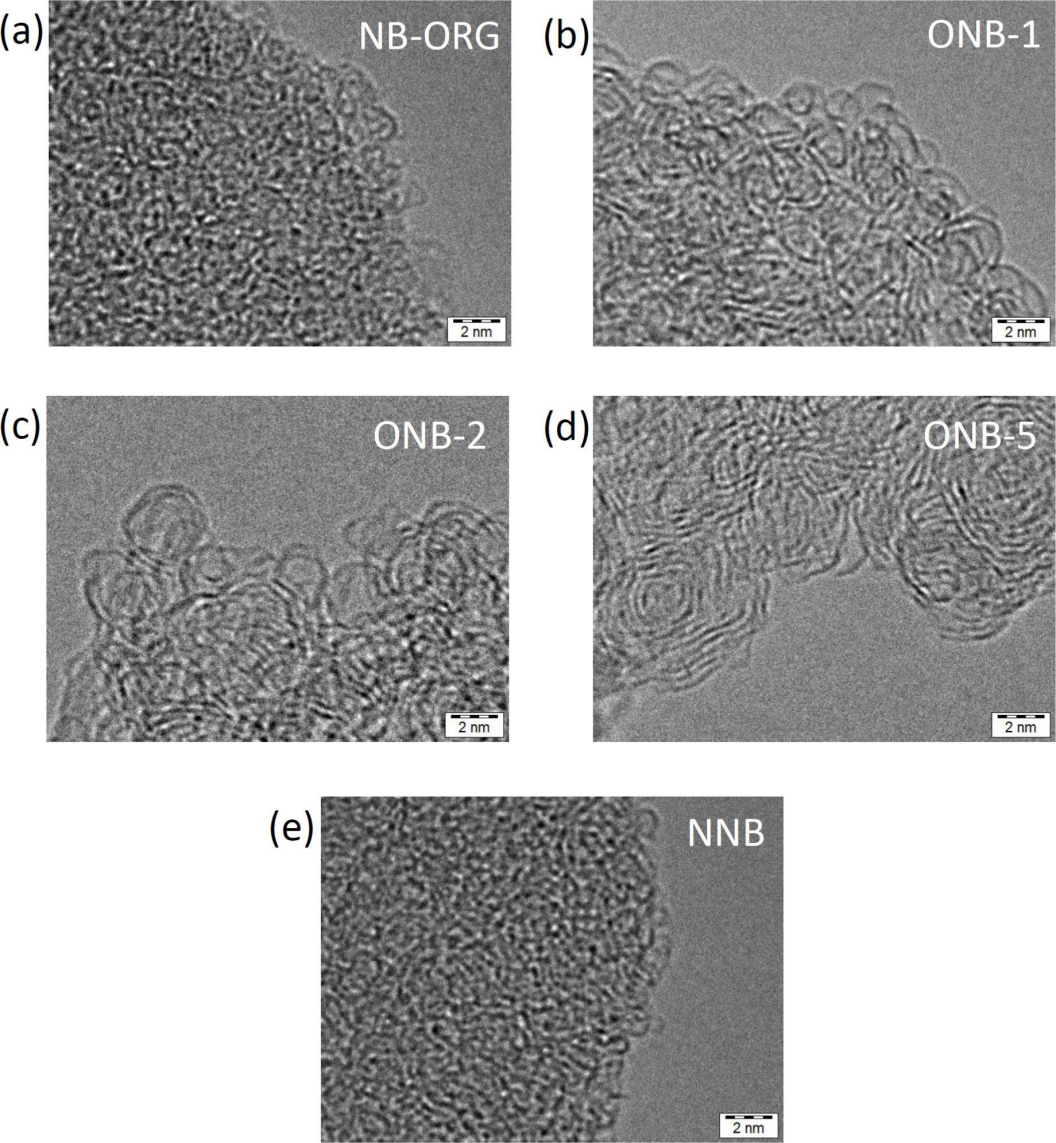
Comparison of transmission electron microscopy (TEM) images of prepared carbons. (a) Unmodified Nanom Black material (NB-ORG), the oxidized samples (b) ONB-1, (c) ONB-2 and (d) ONB-5, and (e) heat-treated sample in an inert nitrogen atmosphere (NNB).

Figure 1 shows selected TEM images of the prepared carbons. NB-ORG did not show a stacking structure, even at higher magnification, but it did show dot-like structures. These results indicate the amorphous nature of this carbon material. WGLs started to appear in ONB-0.5, and they occupied the entire TEM field in ONB-1. Further increases in the oxidation time to 3 h did not yield noticeable changes in the structure of the graphitic layers. The 5 h oxidation resulted in thick graphitic layers forming multilayer onion-like carbons, as shown in Figure 1d. A TEM image of NNB showed similarities to that of NB-ORG, meaning that the heat treatment of NB-ORG in an inert atmosphere did not cause any structural changes under TEM observation.

**Figure 2:**
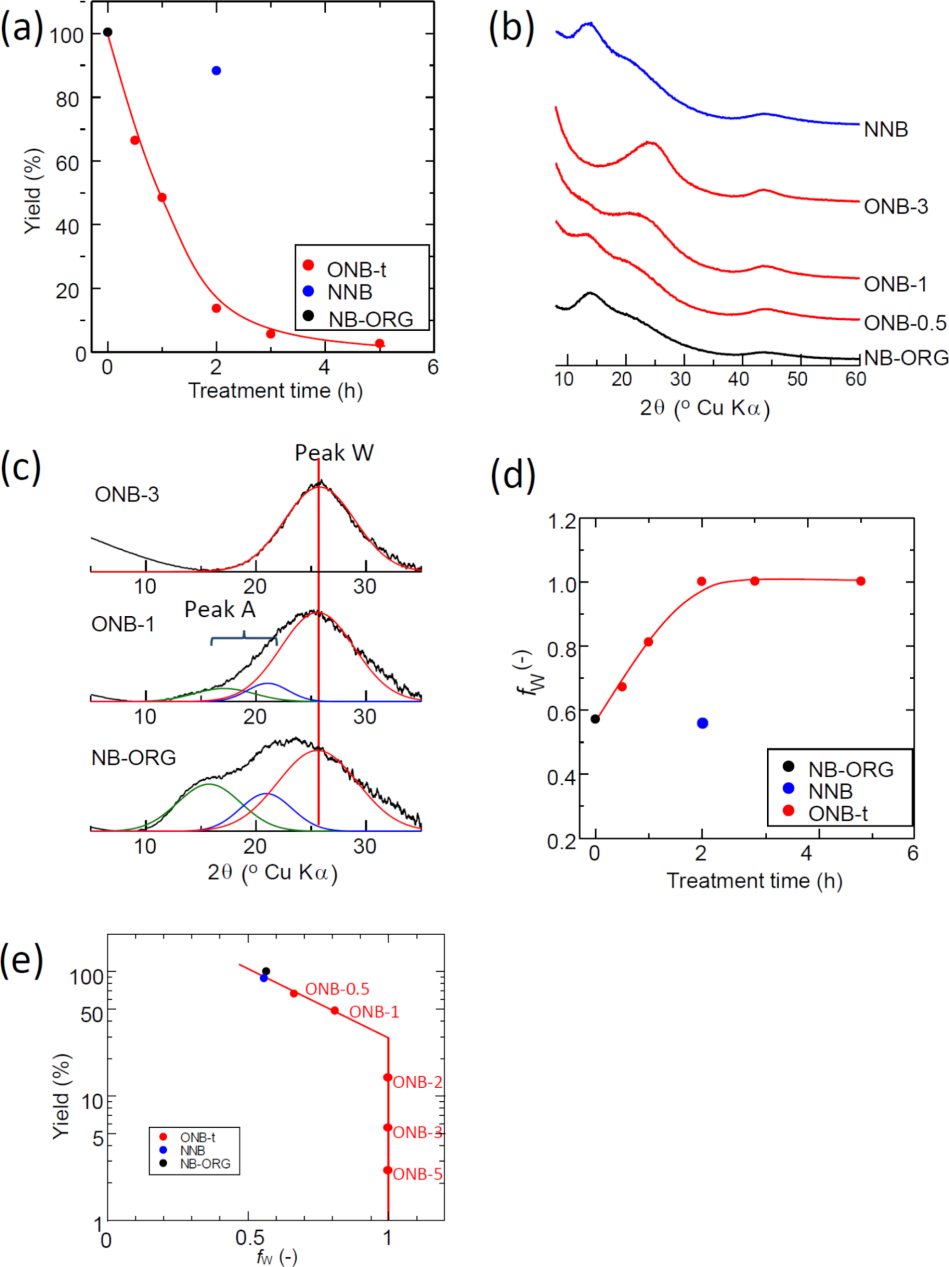
(a) Dependence of the carbon yield on the treatment time. (b) XRD profiles of the prepared carbons. The black line represents the unmodified Namom Black material (NB-ORG), red lines represent the oxidized samples in an O_2_/N_2_ stream (ONB-t series), and the blue line represents the heat-treated sample in a N_2_ stream (NNB). (c) Examples of the peak deconvolution of the 002 XRD profiles of the selected samples; ONB-3, ONB-1 and NB-ORG from top to bottom. The colors of the sub peaks indicate the differences of their origin. The green and blue curves represent amorphous carbon moieties (Peak A) and the red curves the warped graphitic layers (WGLs, Peak W), which were inferred by comparing the XRD profiles and the TEM images shown in Figure 1. (d) Changes of *f*_W_, the fraction of warped graphitic layers (WGLs), calculated as a ratio of the integrated intensity of the peak W in (b) to the total integrated intensity of the 002 region in the vicinity of 002 diffraction with the heat treatment time. (e) Relationship between the yield and *f*_W_.

Figure 2a shows the dependence of the yield on treatment time; there is a rapid decrease in the yield with oxidation time up to 2 h, but no significant decrease for the heat treatment in an inert atmosphere (Figure 2e). Figure 2b shows the XRD profiles of the prepared samples. NB-ORG appeared to have two diffraction peaks in the carbon 002 region: one at 2θ = 17° another at 2θ = 23°. The former was present in other types of fullerene soot, but the origin is not clear [47]. Scanlon et al. reported encapsulated fullerene even in extraction residues [41]. NB-ORG did not show the diffraction characteristics of fullerene crystals, indicating no fullerene nanocrystallites remained in the material. The oxidation treatment to NB-ORG diminished the intensity of the peak at 2θ = 17°, as can be observed in ONB-1 (Figure 2b). This trend was more apparent with extended oxidation treatment time. The peak at around 2θ = 23° became dominant in ONB-2. The results confirm the multicomponent feature of the region in vicinity of 002-diffraction. The diffraction peaks were retrieved by assuming three Gaussian curves. The fraction of the peak at 26.5°, denoted as peak W in Figure 2c, against the total diffraction peaks in this region was defined as *f*_W_. The calculated fraction indicated how dominant the peak W (*f*_W_) is in the material. The change of the fraction *f*_W_ is presented as a function of oxidation time in Figure 2d. The fraction *f*_W_ increased with time up to 2 h of oxidation and then reached unity, as shown in Figure 2d. Comparing this feature with the TEM images, we concluded that the peak W corresponds to the warped graphitic layers. The samples ONB-2, 3, and 5 contained only the structure corresponding to peak W.

### Surface chemical properties

**Figure 3:**
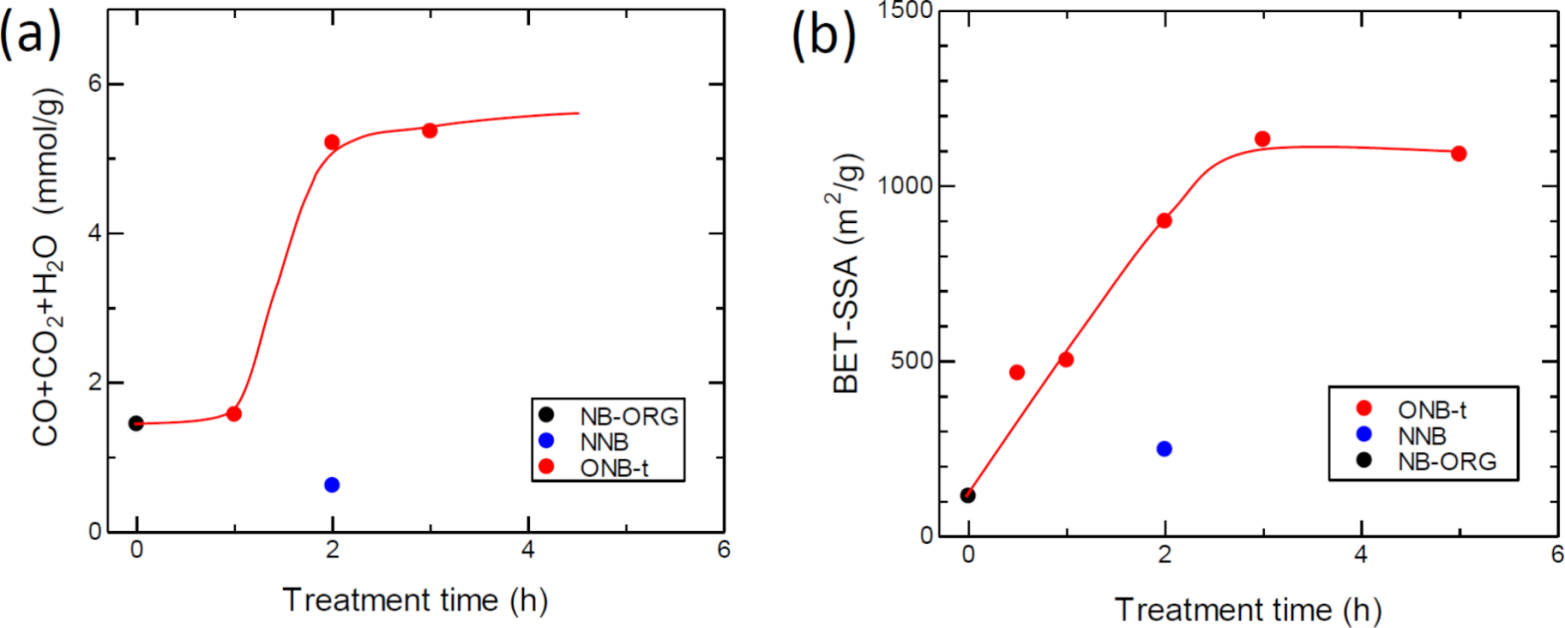
(a) Treatment time dependence of the amount of oxygen-containing surface groups detected by the TPD technique with an upper temperature limit of 1600 °C on the treatment temperature. (b) Dependence of the BET specific surface area of the prepared carbons within the heat-treatment time.

TPD is a useful method to evaluate the amount and type of surface oxygen groups present. Ishii et al. [45–46] extended the analysis temperature up to 1800 °C, which enabled complete desorption of CO-emitting and H_2_-emitting groups, which requires temperatures above 1000 °C. Figure 3a shows the emitted oxygen-containing gases detected by the TPD technique. Oxidation for 1 h did not change the concentration of the surface functional groups, but further oxidation resulted in excess surface oxygen groups and increasing *f*_W_.

**Figure 4:**
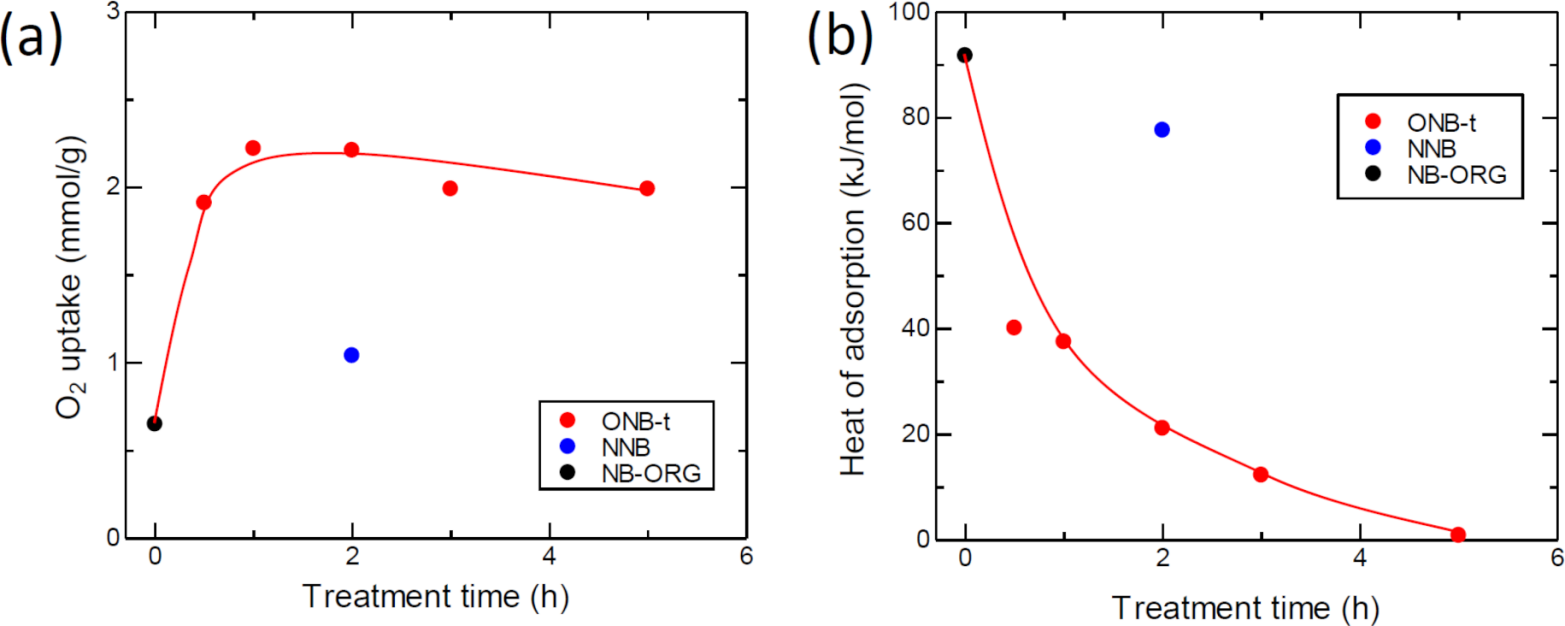
Dependence of the oxygen adsorption properties at −80 °C. (a) O_2_ adsorption uptake measured using a static method. (b) Heat of O_2_-adsorption measured with a dynamic adsorption method using differential scanning calorimetry (DSC).

The techniques used to study the oxygen adsorption properties were a static adsorption technique to measure the amount of adsorbed oxygen and a dynamic adsorption technique to measure the heat of adsorption. Figure 4a and Figure 4b present the changes in the amount of adsorption and the heat of adsorption with oxidation time, respectively. The amount of O_2_ adsorption increased in the first 1 h of oxidation time and then reached saturation. The heat of adsorption showed an abrupt decrease for the 0.5 h oxidation treatment, followed by gradual decrease. NNB did not show such a decrease.

**Figure 5:**
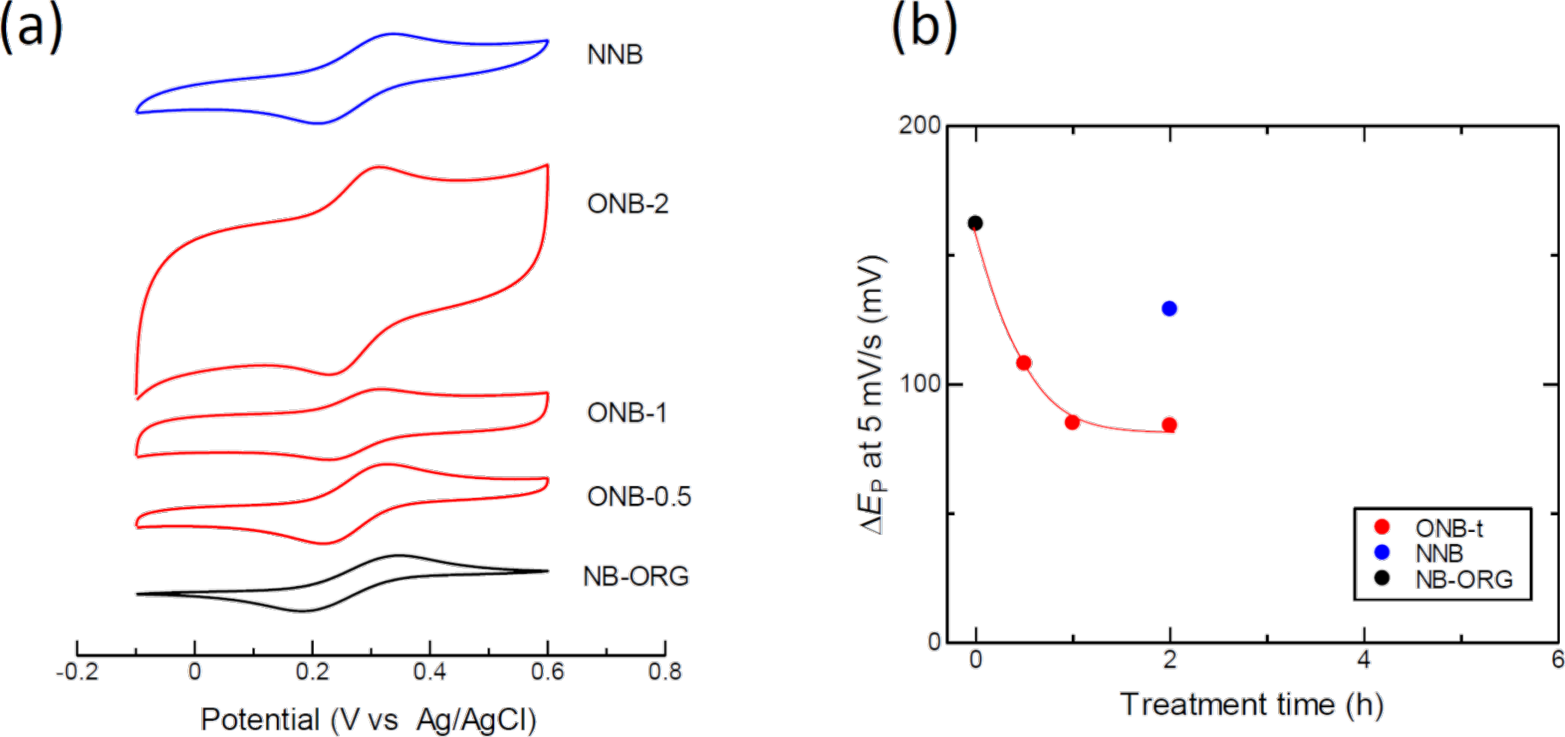
(a) Cyclic voltammograms of the samples for the redox reaction ferricyanide/ferrocyanide (potential sweep rate = 5 mV/s). (b) Dependence of Δ*E*_P_ on treatment time.

Figure 5a shows the cyclic voltammograms of the selected samples using a Fe(CN)_6_^3−^/ Fe(CN)_6_^4−^ redox couple. The cyclic voltammograms showed two peaks, upward (oxidation to Fe(CN)_6_^3−^) and downward (reduction to Fe(CN)_6_^4−^). The potential difference between the oxidation peak and the reduction peak was defined as Δ*E*_P_, an indicator of the heterogeneous electron transfer rate. When the Δ*E*_P_ value is close to 57 mV, the system is reversible, meaning that the electron transfer at the interface between the electrode and the electrolyte is very high. On the other hand, the increases in Δ*E*_P_ correspond to slow electron transfer. Figure 5b shows a plot of Δ*E*_P_ as a function of oxidation time; the Δ*E*_P_ value decreased rapidly with oxidation time up to 1 h. NNB did not show such a decrease in Δ*E*_P_. The oxidized carbons, ONBs, showed smaller values than NB-ORG and NNB, indicating an accelerated electron transfer process.

### ORR catalytic activity

**Figure 6:**
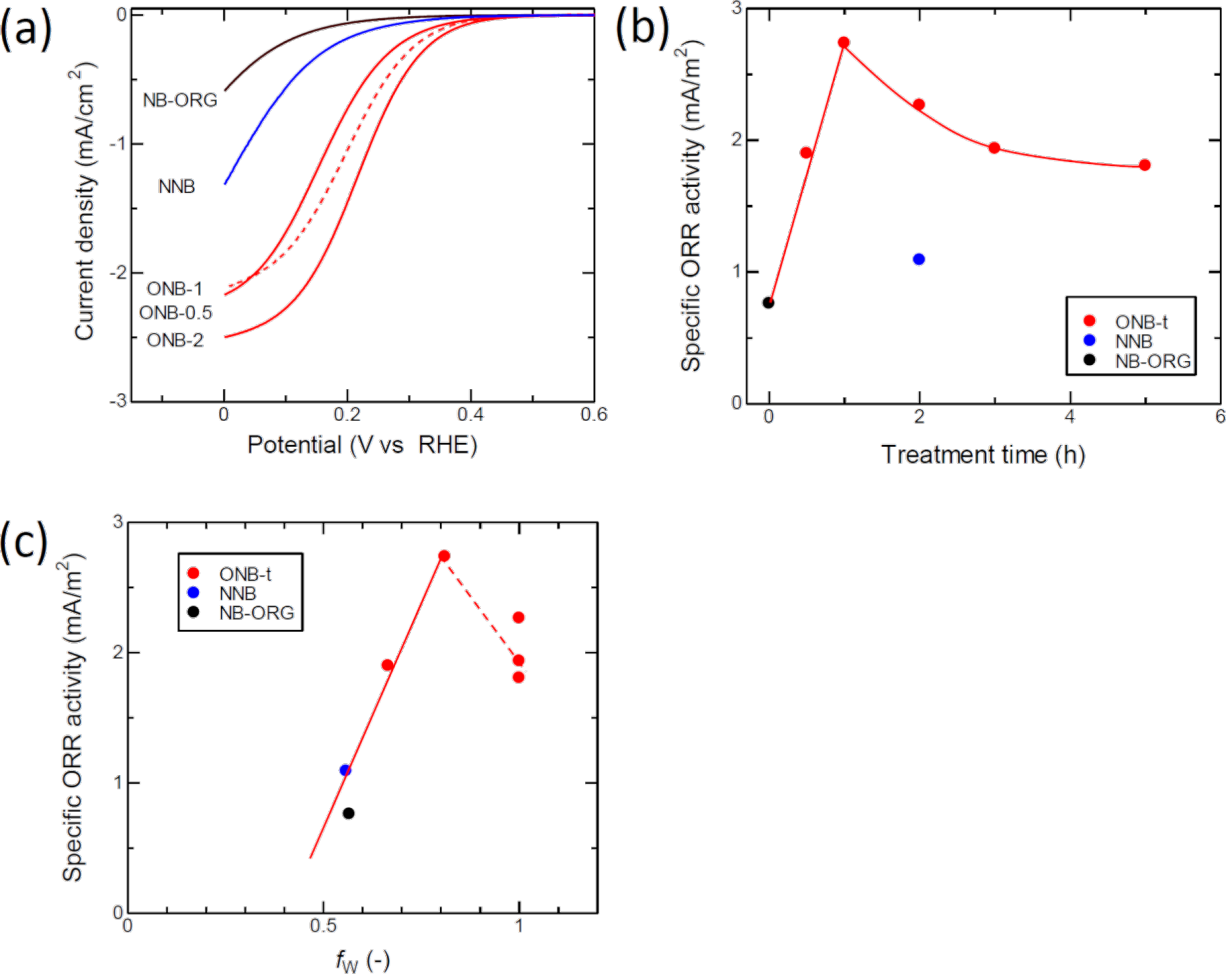
(a) Oxygen reduction reaction (ORR) voltammogram in 0.5 mol/L H_2_SO_4_ solution. (b) Dependence of specific ORR activity on treatment time. (c) Relationship between the specific ORR activity and *f*_W_.

Figure 6a presents the ORR voltammograms of the prepared carbons obtained in a 0.5 mol/L H_2_SO_4_ solution. NB-ORG showed the lowest ORR activity, as can be recognized from the lowest onset potential and the lowest reduction current. The oxidation treatment increased the ORR activity, as observed in the voltammograms of ONBs. NNB showed slight increases in both the onset potential and current; however, its magnitude was lower than that of the ONBs. Figure 6b shows the change in the specific ORR activity as a function of oxidation time, where the specific ORR activity was a current density of 0.3 V vs RHE normalized by the corresponding BET-SSA. The ORR activity increased with the oxidation time for the first 1 h and then decreased. Figure 6c shows a plot of the specific ORR activity against *f*_W_. The specific ORR activity of the ONBs prepared for an oxidation time of less than 2 h correlated well with *f*_W_.

## Discussion

### Formation of WGLs from NB-ORG by oxidation

We initially expected NB-ORG to include WGLs because of the incomplete formation of the fullerene structure during the manufacturing process. If we can separate WGLs from NB-ORG, the extracted WGLs should be a well-defined model to study the origin of the ORR activity of CACs. We postulated the selective oxidation of amorphous moieties in NB-ORG to separate WGLs. TEM observations confirm the formation of WGLs through the oxidation by removing the amorphous moieties. The changes in the shape of the XRD profiles with treatment also supports our theory. The relationship between the oxidation treatment yield and *f*_W_ calculated from the XRD profiles in the vicinity of 002 diffraction illuminates the structural changes with the progression of oxidation. First, amorphous carbons reacted with oxygen to produce and remove CO_2_, leaving WGLs behind. Further oxidation results in the formation of oxygen functional groups on WGLs, as observed by TPD. The oxidation treatment also increases the BET-SSA, which corresponds to the removal of amorphous carbon moieties. The results confirm that NB-ORG originally included WGLs embedded in amorphous moieties. The oxidation treatment resulted in WGLs by the selective oxidation of amorphous moieties. Furthermore, chemical analysis of NB-ORG confirmed the absence of the foreign atoms that are effective at enhancing ORR activity. Therefore, the obtained WGLs were also free from such foreign elements.

Oxygen appears to play an important role in forming the five-membered rings that are necessary to construct WGLs. The reaction of fullerenes with oxygen molecules at elevated temperatures induces cage opening [48–50]. Furthermore, some oxygen compounds can form orifices or holes on fullerene molecules; this is recognized as an important synthesis technique for “molecular surgery” [51]. Blending a biodiesel oil containing oxygen atoms into an ordinary diesel oil resulted in the formation of soot with WGLs [52]; the authors explain this phenomenon by the C_5_-forming ability of oxygen included in the biodiesel oil. These two studies reported on the formation of WGLs in commonly included reactions between oxygen and carbon atoms. The results indicate the possibility of that WGLs in the present study formed during the oxidation. The NB-ORG used in this study already included WGLs, as evidenced by the multicomponent feature of the XRD profiles in the vicinity of 002 diffraction, which was supported by TEM observation. Ultimately, the formation of the WGLs by the oxidation of NB-ORG occurred by removing amorphous carbons through oxidation.

### Factors determining ORR catalytic activity of carbons

We have shown that the oxidation treatment of NB-ORG brought about the formation of WGLs, the enhancement of the ORR activity, an increase in the specific surface area, and an increase in surface oxygen functional groups. Herein, we discuss the specific ORR activity defined in the previous section, which eliminates the influence of the BET-SSA on the ORR activity. The specific activity increased with oxidation time up to 1 h and then decreased, as shown in Figure 3b. The increasing behavior of the specific activity for the first 1 h agrees with the development of the WGL phase, which is confirmed by the correlation between the specific activity and *f*_W_ (Figure 6c). However, the correlation was not maintained for samples with *f*_W_ ≈ 1. This initially seems to disprove our assumption, because the result indicates that the pure WGLs had less activity than the less pure WGLs. Thus, we consider another parameter, the surface concentration of the oxygen surface functional groups. We measured the surface oxygen concentration by using a TPD technique. The obtained concentration of the surface oxygen functional groups increased suddenly between 1 and 2 h of oxidation time. From these results, it can be seen that longer oxidation times result in the two opposite effects on specific ORR activity: the development of WGLs and oxidation to introduce oxygen functional groups to WGLs.

Surface oxidation of carbon is reported to deteriorate ORR activity. Banham et al. studied the degradation of non-precious-metal catalysts and a carbon alloy catalyst [38]. They claim that carbon oxidation (attacked by H_2_O_2_) is the primary mechanism for performance loss during cell operation. Hence, this conclusion justifies the degrading effect of the specific activity observed for the intensively oxidized NBs. Finally, we have two competing factors to control the ORR activity of carbon with the oxidation treatment – the development of the WGL phase as the promoting factor and the increase in the concentration of surface oxygen groups as the inhibiting factor. The changes in these two factors finally determined the specific ORR activity of the ONBs.

Next, we considered the reason for the ORR activity increase with the development of the WGL phase. Removing the amorphous phase by the oxidation treatment exposed the WGL to the material surface and to contact with the reactants, O_2_ and protons. The exposure of the WGL phase by removing the amorphous phase meant a change in the surface property from that governed by the amorphous carbon phase to that governed by the WGL phase. The ORR activity change was a result of the surface change due to the exposure of the WGL phase to the surface of the material; hence, we considered the chemical characteristics of the WGL-exposed NBs (ONBs) by paying special attention to ONBs that did not experience the introduction of oxygen surface groups (i.e., the ONBs obtained by the oxidation for shorter than 1 h).

The oxygen adsorption uptake and heat of oxygen adsorption varied with oxidation time. The oxidation of NB-ORG immediately increased the O_2_ adsorption and decreased the heat of adsorption. The increase in adsorption uptake accelerates the following elementary step through the concentration term in the rate equation. The decrease in the heat of adsorption activated the adsorbed state of oxygen molecules. However, lowering the heat too much led to a small interaction between the adsorbent and the adsorbate (i.e., less activation).

Further evidence for the changes in the surface properties due to WGL exposure is the change in the electron transfer rate evaluated by the Δ*E*_P_ of Fe(CN)_6_^3−^/Fe(CN)_6_^4−^ cyclic voltammograms. The Δ*E*_P_ value decreased rapidly in the first 2 h of oxidation, approaching the limit of the fastest electron transfer, Δ*E*_P_ = 57 mV. This behavior confirms that the exposure of WGLs to the surface of the material enhances electron transfer. Another interesting point is that the electron transfer was not inhibited by the oxygen surface functional groups. This may be because the redox reaction does not require any adsorption sites, unlike ORR.

## Conclusion

In summary, we obtained WGLs by the oxidation of Nanom Black (NB-ORG), a fullerene extraction residue, at 600 °C and tested our assumptions about the ORR activity of WGLs by examining their structure, properties, and ORR activity. First, we clarified the mechanism of the WGL formation from NB-ORG as the removal of an amorphous carbon matrix surrounding the originally included WGLs. The specific ORR activity, defined as an ORR current normalized by the corresponding BET surface area, showed a maximum when the oxidation time was 1 h. The presence of the maximum activity was understood in terms of two competing factors, the development of WGLs and the increase in oxygen surface functionality with oxidation time. The role of WGLs in ORR was also found to lower the heat of O_2_-adsorption to a suitable value for O_2_ activation and to accelerate heterogeneous electron transfer. The present study highlights important considerations for the design of non-metal carbon-based cathode catalysts for PEFCs and illuminates interesting aspects of carbon materials.

## References

[1] Banham D, Choi J-Y, Kishimoto T, Ye S (2019). Adv Mater (Weinheim, Ger).

[2] Toda T, Igarashi H, Watanabe M (1999). J Electroanal Chem.

[3] Zhang J, Lima F H B, Shao M H, Sasaki K, Wang J X, Hanson J, Adzic R R (2005). J Phys Chem B.

[4] Inoue H, Ishii T, Kannari N, Ozaki J-i (2016). ChemistrySelect.

[5] Jasinski R (1964). Nature.

[6] Jahnke H, Schönborn M, Zimmermann G (1976). Organic dyestuffs as catalysts for fuel cells. Physical and Chemical Applications of Dyestuffs.

[7] Lefèvre M, Dodelet J P, Bertrand P (2002). J Phys Chem B.

[8] Lefèvre M, Proietti E, Jaouen F, Dodelet J-P (2009). Science.

[9] Maldonado S, Stevenson K J (2005). J Phys Chem B.

[10] Ozaki J-i, Kimura N, Anahara T, Oya A (2007). Carbon.

[11] Razmjooei F, Singh K P, Song M Y, Yu J-S (2014). Carbon.

[12] Li R, Wei Z, Gou X (2015). ACS Catal.

[13] Gao J, Ma N, Tian J, Shen C, Wang L, Yu P, Chu Y, Liu W, Tan X, Li X (2018). J Solid State Electrochem.

[14] Huang S-F, Terakura K, Ozaki T, Ikeda T, Boero M, Oshima M, Ozaki J-i, Miyata S (2009). Phys Rev B.

[15] Ozaki J-i, Anahara T, Kimura N, Oya A (2006). Carbon.

[16] Ikeda T, Boero M, Huang S-F, Terakura K, Oshima M, Ozaki J-i, Miyata S (2010). J Phys Chem C.

[17] Matter P H, Wang E, Arias M, Biddinger E J, Ozkan U S (2007). J Mol Catal A: Chem.

[18] Gong K, Du F, Xia Z, Durstock M, Dai L (2009). Science.

[19] Mamtani K, Jain D, Dogu D, Gustin V, Gunduz S, Co A C, Ozkan U S (2018). Appl Catal, B.

[20] Qu K, Zheng Y, Dai S, Qiao S Z (2016). Nano Energy.

[21] You J-M, Ahmed M S, Han H S, Choe J e, Üstündağ Z, Jeon S (2015). J Power Sources.

[22] Bag S, Mondal B, Das A K, Raj C R (2015). Electrochim Acta.

[23] Jiang H, Yao Y, Zhu Y, Liu Y, Su Y, Yang X, Li C (2015). ACS Appl Mater Interfaces.

[24] Zhang X, Lin J, Chen S, Yang J, Song L, Wu X, Xu H (2017). ACS Appl Mater Interfaces.

[25] Kim B J, Lee D U, Wu J, Higgins D, Yu A, Chen Z (2013). J Phys Chem C.

[26] Varnell J A, Tse E C M, Schulz C E, Fister T T, Haasch R T, Timoshenko J, Frenkel A I, Gewirth A A (2016). Nat Commun.

[27] Tan H, Li Y, Kim J, Takei T, Wang Z, Xu X, Wang J, Bando Y, Kang Y-M, Tang J (2018). Adv Sci.

[28] Wu Z-Y, Xu X-X, Hu B-C, Liang H-W, Lin Y, Chen L-F, Yu S-H (2015). Angew Chem, Int Ed.

[29] Ozaki J, Imashiro Y (2015). Electrochemistry.

[30] Ozaki J-i, Nozawa K, Yamada K, Uchiyama Y, Yoshimoto Y, Furuichi A, Yokoyama T, Oya A, Brown L J, Cashion J D (2006). J Appl Electrochem.

[31] Ozaki J-i, Tanifuji S-i, Furuichi A, Yabutsuka K (2010). Electrochim Acta.

[32] Ishii T, Maie T, Kimura N, Kobori Y, Imashiro Y, Ozaki J-i (2017). Int J Hydrogen Energy.

[33] Kannari N, Ozaki J-i (2012). Carbon.

[34] Kannari N, Takigami M, Maie T, Honda H, Kusadokoro S, Ozaki J-i (2013). Smart Grid Renewable Energy.

[35] Maie T, Ozaki J-i (2016). J Electrochem Soc.

[36] Ishii T, Maie T, Hamano M, Kishimoto T, Mizushiri M, Imashiro Y, Ozaki J-i (2017). Carbon.

[37] Banham D, Kishimoto T, Sato T, Kobayashi Y, Narizuka K, Ozaki J-i, Zhou Y, Marquez E, Bai K, Ye S (2017). J Power Sources.

[38] Banham D, Kishimoto T, Zhou Y, Sato T, Bai K, Ozaki J-i, Imashiro Y, Ye S (2018). Sci Adv.

[39] Kannari N, Itakura T, Ozaki J-i (2015). Carbon.

[40] Takehara H, Fujiwara M, Arikawa M, Diener M D, Alford J M (2005). Carbon.

[41] Scanlon J C, Ebert L B (1993). J Phys Chem.

[42] Grieco W J, Howard J B, Rainey L C, Vander Sande J B (2000). Carbon.

[43] Kannari N, Nakamura Y, Ozaki J-i (2013). Carbon.

[44] Iwashita N, Park C R, Fujimoto H, Shiraishi M, Inagaki M (2004). Carbon.

[45] Ishii T, Kashihara S, Hoshikawa Y, Ozaki J-i, Kannari N, Takai K, Enoki T, Kyotani T (2014). Carbon.

[46] Ishii T, Kaburagi Y, Yoshida A, Hishiyama Y, Oka H, Setoyama N, Ozaki J-i, Kyotani T (2017). Carbon.

[47] Werner H, Herein D, Blöcker J, Henschke B, Tegtmeyer U, Schedel-Niedrig T, Keil M, Bradshaw A M, Schlögl R (1992). Chem Phys Lett.

[48] Wohlers M, Bauer A, Rühle T, Neitzel F, Werner H, Schlögl R (1997). Fullerene Sci Technol.

[49] Chen H S, Kortan A R, Haddon R C, Kaplan M L, Chen C H, Mujsce A M, Chou H, Fleming D A (1991). Appl Phys Lett.

[50] Wohlers M, Werner H, Herein D, Schedel-Niedrig T, Bauer A, Schlögl R (1996). Synth Met.

[51] Gan L, Yang D, Zhang Q, Huang H (2010). Adv Mater (Weinheim, Ger).

[52] Vander Wal R L, Strzelec A, Toops T J, Stuart Daw C, Genzale C L (2013). Fuel.

